# Endometrial stromal cells exhibit a distinct phenotypic and immunomodulatory profile

**DOI:** 10.1186/s13287-019-1496-2

**Published:** 2020-01-06

**Authors:** Suzanna Queckbörner, Elisabeth Syk Lundberg, Kristina Gemzell-Danielsson, Lindsay C. Davies

**Affiliations:** 1 Department of Women’s and Children’s Health, Division of Obstetrics and Gynecology, Karolinska Institutet, and Karolinska University Hospital, S-171 64 Solna, Sweden; 2 Department of Clinical Genetics, Karolinska University Hospital, S-171 76 Stockholm, Sweden; 3 Department of Laboratory Medicine, Karolinska Institutet, S-141 52 Huddinge, Sweden

**Keywords:** Endometrium, Stroma, Immunomodulation, Cell therapy, T cell, Asherman’s syndrome, Inflammation, Infertility, Mesenchymal stromal cell

## Abstract

**Background:**

In Asherman’s syndrome (AS), intrauterine scarring and fibrotic adhesions lead to menstrual disorders, pregnancy loss, or infertility. A few clinical trials have piloted cell therapy to overcome AS. Understanding the role of the stromal compartment in endometrial regeneration remains poorly understood. We hypothesize that endometrial stromal cells (eSCs) represent a relevant cell population to establish novel cell-based therapeutics for endometrial disorders. The aim of this study was to characterize eSCs and evaluate their immune-cell interactions.

**Methods:**

eSCs were isolated from healthy donors, during the proliferative stage of the menstrual cycle. Cells were characterized for expression of mesenchymal stromal cell (MSC) markers and assessed for their tumorigenic potential. eSCs were co-cultured with interferon γ and tumor necrosis factor α, and cell surface expression of their respective receptors and human leukocyte antigen (HLA) I and II determined by flow cytometry. Secreted levels of key immunomodulatory factors were established. eSCs were cultured with activated peripheral blood mononuclear cells, and T cell differentiation and proliferation determined.

**Results:**

eSCs demonstrated an MSC surface phenotype and exhibited multipotency. Expanded eSCs retained chromosomal stability and demonstrated no tumorigenicity. Upon stimulation, eSCs licensed to an anti-inflammatory phenotype with upregulated secretion of immunomodulatory factors. Stimulated eSCs did not express HLA class II. eSCs suppressed the proliferation and activation of CD4+ T cells, with the eSC secretome further downregulating central memory T cells and upregulating effector memory (EM) cells.

**Conclusions:**

Differential responsiveness to inflammation by eSCs, compared to other MSC sources, demonstrates the need to understand the specific functional effects of individual stromal cell sources. A lack of HLA class II and triggering of EM T cell differentiation strongly links to innate in vivo roles of eSCs in tissue repair and immune tolerance during pregnancy. We conclude that eSCs may be an ideal cell therapy candidate for endometrial disorders.

## Background

Healthy endometrium regenerates approximately 450 times in a women’s reproductive life without the formation of scar tissue [1]. The endometrium makes up the inner lining of the uterus with 2 distinct layers, the *basalis* and the transient *functionalis*. In a hormonally regulated 28-day cycle, the functional layer proliferates, differentiates, secretes, disintegrates, and repairs following menses. It has been hypothesized that this process of endometrium regeneration is orchestrated, in part, by an endometrial progenitor cell population [2, 3]. Deregulation of this cycle is seen in endometrial pathologies such as Asherman’s syndrome (AS), where patients can suffer from endometrial scarring, fibrosis, and adhesions occluding the uterine cavity [4].

The menstrual cycle and its 3 main stages, menstruation and the proliferative and secretory phases, have been likened to the phases of wound repair [5–7]. Classical wound healing, as seen in the dermis, is accompanied by the formation of tissue granulation and scar maturation [8]. However, in healthy endometrium, no scarring is observed, suggesting regenerative healing, more akin to the fetal-like response seen in the buccal mucosa of the oral cavity [9–12].

The stromal compartment makes up the largest proportion of the endometrium and controls tissue proliferation, remodeling, and breakdown during the menstrual cycle, under the tight regulation of estrogen and progesterone, and local and peripheral immune cells [13]. Immune cell activation, abundance, and distribution vary throughout the menstrual cycle. Earlier research has investigated how the immune-profile of macrophage, neutrophil, T cell, and NK cell compartments change in the context of menses, pregnancy, and to a lesser extent healthy endometrial regeneration [14–17]. Flynn et al. [17] demonstrated that the frequency and ratio of T cell subsets differs during the proliferative and secretory phases of the menstrual cycle, suggesting dynamic immune-stromal cell relationships in tissue homeostasis [17]. However, few human studies have sought to further explore stromal-immune cross-talk, a factor demonstrated to be of central importance in the context of stromal cell therapies, and in the context of tissue regeneration, where the presumed mechanism of action is modulation of the local immune environment [18].

Endometrial research to date has primarily focused on factors regulating embryo implantation, emphasizing the importance of decidualization and placentation in fertility and pregnancy success. Understanding of the endometrial stromal compartment’s role in regeneration after menstrual shedding remains scarce. Further knowledge will be critical in developing a better biological understanding of benign, fibrotic gynecological diseases such as AS and endometriosis, with the aim of developing suitable therapies. Cell therapy has been a steadily expanding field with 327 active clinical trials, registered with clinicaltrials.gov (as of October 28, 2019), involving “mesenchymal cells.” These cell products have been primarily developed to alleviate diseases with pro-inflammatory components, or a need to repair damaged tissue. This growing trend towards cell therapeutics has now been proposed and piloted for endometrial restoration [19–23].

In the last 10 years, stromal cells derived from menstrual blood (menSCs) and umbilical cord (UC MSCs) have been piloted as a cell therapy for infertility and AS (reviewed [23]). These pilot studies have provided promising results, with increased endometrial thickness (an established measurement of responsiveness to therapy) and temporarily resumed menstruation [21, 24]. With the development of cellular therapeutics, especially within the area of MSC therapies, there has been increasing recognition of the need to understand the mechanism of action of heterogeneous cell sources and to move away from the concept of “one cell fits all,” thereby acknowledging the tissue of origin when establishing a therapeutic. This is exemplified with recent single-cell RNA-sequencing studies showing that decidualized endometrial stromal cells have a specific gene expression and immune cell modulation relative to their tissue origin [25]. Equally, although MenSCs are of endometrial origin, their immune phenotype has been suggested to be more inflammatory due to the phenotypic changes caused by exposure to tissue breakdown products and apoptosis [5, 26, 27]. We therefore hypothesize that healthy, proliferative phase endometrium would represent a better alternative for cell therapy development in endometrial restoration, including the treatment of AS.

The aim of this study was to provide a detailed characterization of the endometrial stromal compartment in the proliferative phase of the menstrual cycle. In the context of cell therapy, we have sought to determine whether endometrial stromal cells (herein termed eSCs) can be classified as MSCs according to the International Society of Cellular Therapy (ISCT) minimal criteria [28, 29], determine their tumorigenic potential in vitro, and establish their immunomodulatory capacity in response to pro-inflammatory signals.

## Methods

### Donor material

Endometrial samples were obtained, during the proliferative stage of the menstrual cycle, from healthy women (*n* = 6), aged 24–32 years. The study was approved by the regional ethics committee of Karolinska Institutet, Stockholm, Sweden, and conducted in line with the Helsinki Declaration. Written informed consent was obtained from all participating women.

All donors had normal menstrual cycles (25–35 days) and were proven fertile (at least one confirmed pregnancy). Donors were examined for the absence of hormonal diseases, uterine pathologies (e.g., endometriosis, polycystic ovary syndrome and/or previous infertility records), and sexually transmitted diseases (human immune deficiency disease, *Chlamydia trachomatis*-DNA, and Gonococci-DNA). None of the women had used hormonal contraception or an intrauterine device for a minimum of 3 months prior to biopsy. The subject’s proliferative stage (cycle day [CD] 7–9) was calculated based on their previous time of menstruation. The biopsies were obtained from the functional layer of the endometrium, without cervical dilation or local anesthesia, using a pipelle aspirator from Cooper Surgical (Trumbull, USA).

### Endometrial stromal cell isolation and maintenance in culture

The biopsy was washed and finely minced in Minimum Essential Medium α (MEM α; Thermo Fisher Scientific, Dreieich, Germany) with 5% (v/v) pooled human platelet-lysate (Karolinska University Hospital, Huddinge, Sweden), 100 IU/ml penicillin, 0.1 mg/ml streptomycin (Thermo Fisher Scientific), and 20 μl/ml heparin 1000 IE/ml (APL, Stockholm, Sweden; complete media), before digesting with dispase II (0.75 U/ml; Sigma-Aldrich, Taufkirchen, Germany) in complete media with agitation for 45 min at 37 °C/5% CO_2_. Isolated cells were strained through a nylon mesh 70-μm cell strainer (Corning, Gothenburg, Sweden) and incubated at 37 °C/5% CO_2_ in 1X TrypLE™ Express Enzyme (Thermo Fisher Scientific) for 10 min, followed by washing with complete media and a centrifugation (400×*g*) step. Resuspended cells were plated at a density of 1E+04 cells/cm^2^ in complete media. When adherent eSCs reached confluence (> 80%), the cells were detached in TrypLE™ and replated at the above density. All cells were checked for mycoplasma contamination using the EZ-PCR™ Mycoplasma Test Kit (Biological Industries, Cromwell, USA) as per the manufacturer’s instructions.

### Colony-forming unit-fibroblast capacity and growth kinetics

The number of colony-forming unit-fibroblasts (CFU-F) relative to the number of seeded eSCs after plastic adherence and expansion for one passage was recorded. Briefly, 1E+02 eSCs, at passage 1 (P1; *n* = 6), were seeded into 1 well of a 6-well plate. Cells were cultured in complete media for 14 days, with media changes every 3 days. Cells were washed with phosphate-buffered saline (PBS; Sigma-Aldrich) and fixed with 70% (v/v) ethanol (Histolab, Gothenburg, Sweden) for 60 min, before washing with PBS and staining with 0.1% (w/v) crystal violet (Sigma-Aldrich) dissolved in distilled water. Colonies of more than 32 cells were counted allowing exclusion of transit amplifying cells [30]. CFU-F was calculated using the below formula:

$$ \mathrm{CFU}-F=\frac{\mathrm{number}\ \mathrm{of}\ \mathrm{colonies}\ \mathrm{formed}}{\mathrm{number}\ \mathrm{of}\ \mathrm{cells}\ \mathrm{seeded}}\times 100 $$

Growth kinetics were monitored by calculating cell population doublings (PDs) at each passage using the formula log *n*/log 2, where *n* is the number of cells at harvest, divided by the number of cells seeded. The cumulative PD, up to P6, was calculated to evaluate the proliferative capacity of each donor. PD rate was calculated by dividing the number of days in culture with the number of PDs recorded.

### Characterization of endometrial stromal cell surface markers

The MSC phenotype of passaged cells at P3-4 was confirmed by flow cytometry as per the ISCT guidelines (*n* = 6) [28]. eSCs were detached from culture flasks using 0.05% (v/v) Trypsin-EDTA (Thermo Fisher Scientific) and evaluated for cell surface expression of CD73, CD90, CD105, CD14, CD19, CD34, CD45, and human leukocyte antigen (HLA) I and II (see Table 1 for details of antibodies and IgG controls). LIVE/DEAD™ Fixable Aqua Dead Cell Stain Kit (Thermo Fisher Scientific) was used to assess viability. All cells were stained for 15 min in the dark at room temperature, before washing in PBS, centrifugation at 400×*g* for 5 min and resuspension in PBS/0.1% (w/v) bovine serum albumin (BSA, Sigma-Aldrich) for analysis. Samples were run on a CytoflexS flow cytometer (Beckman Coulter, Bromma, Sweden) equipped with 4 lasers: 405 nm, 638 nm, 488 nm, and 561 nm, and 10,000 gated events recorded. Data was analyzed using FlowJo software (v10.6.1; BD, OR, USA).

**Table 1:** Antibodies and Ig controls used for cell surface characterization

Antibodies/isotype controls	Clone	Fluorochrome	Company	Catalog number
CD90	5E10	PERCP Cy5.5	Biolegend	328117
CD73	AD2	APC	Abcam	ab155378
CD105	MEM-229	PE	Abcam	ab53321
CD45	HI30	PB	Biolegend	304021
CD14	HCD14	PB	Biolegend	325615
CD19	HIB19	PB	Biolegend	302223
CD34	581	PB	Biolegend	343511
CD120a	W15099A	APC	Biolegend	369906
CD120b	REA520	PE	Miltenyi Biotec	130107705
CD119	GIR-94	PE	Biolegend	308703
HLA II	CR3/43	FITC	Dako	F0817
HLA I	W6/32	AF 488	Biolegend	311413
CD3	UCHT1	V450	BD Bioscience	560365
CD4	RPA-T4	PERCP Cy5.5	BD Bioscience	560650
CD25	M-A251	PE	BD Bioscience	555432
CD27	M-T271	AF700	BD Bioscience	560611
CD127	HIL-7R-M21	AF647	BD Bioscience	558598
CD45RA	HI100	PE	Biolegend	304107
Mouse IgG1 /IgG2a		PE/FITC	BD Bioscience	342409
Mouse IgG1, κ	15H6	APC	Abcam	ab37391
Mouse IgG1, κ	MOPC-21	PB	Biolegend	400131
Mouse IgG1, κ	MOPC-21	PerCP Cy5.5	Biolegend	400149
Mouse IgG1, κ	11711	PE	R&D Systems	IC002P
Mouse IgG2a	X5563	PE	Abcam	ab91363

*APC* allophycocyanin, *AF* Alexa Fluor, *PB* Pacific Blue, *FITC* fluorescein isothiocyanate, *PE* phycoerythrin, *PerCP Cy5.5* peridinin chlorophyll protein

### Osteogenic and adipogenic differentiation

eSCs were evaluated for their multipotency by differentiating down osteogenic and adipogenic lineages using StemMACS™ OsteoDiff Media (Miltenyi Biotec, Bergisch Gladbach, Germany) and StemPro™ Adipogenesis Differentiation Kit (Thermo Fisher Scientific) respectively. Differentiation was performed according to the manufacturer’s instructions. Briefly, 1E+05 eSCs at P2 were seeded in 1 well of a 12-well plate and cultured in complete media until reaching 100% confluence (*n* = 5). The cells were then cultured for 21 days with differentiation media, with media changed every 3 days. The control wells continued to be treated with complete media. Differentiation was assessed using *Alizarin* Red (osteogenic) and Oil Red O (adipogenic) staining.

#### Alizarin Red staining

Cells were washed and fixed in 10% (v/v) formalin (Histolab) and stained with 2% (w/v) *Alizarin* Red solution (Sigma-Aldrich), pH 4.1–4.3, for 20 min before washing with distilled water. Cells were imaged using a Nikon Eclipse Ts 2 microscope (Nikon, Solna, Sweden) using IC Measure Software v2.0.0133 (The Imaging Source Europe, Bremen, Germany).

#### Oil Red O staining

Cells were washed twice with PBS and fixed with 10% (v/v) formalin for 60 min at room temperature. Fixed cells were rinsed with distilled water and incubated with 60% (v/v) 2-Propanol (Sigma-Aldrich) for 5 min at room temperature. Cells were stained with 0.6% (w/v) Oil Red O (Sigma-Aldrich) for 10 min at room temperature before washing with distilled water.

### Karyotyping

eSCs (*n* = 4, P3) were karyotyped by G-banding at the Department of Clinical Genetics, Karolinska University Hospital, Solna, Sweden. eSCs were cultured to 60% confluence and treated with 10 μg/mL KaryoMAX™ Colcemid™ Solution (Thermo Fisher Scientific) for 5 h, followed by dissociation with TrypLE™. The cells were pelleted via centrifugation at 140×*g* for 10 min, resuspended in pre-warmed hypotonic solution (1:2 0.56% (w/v) potassium chloride: 0.6% (w/v) sodium citrate; Sigma-Aldrich), and incubated for 10 min at 37 °C/ 5% CO_2_. Following centrifugation, the cells were resuspended in fixative (3:1 methanol: acetic acid; Sigma-Aldrich) at room temperature. Metaphase spreads were prepared on glass microscope slides and G-banded by brief exposure to 0.017% trypsin (Thermo Fisher Scientific) in PBS and stained with Gurr’s/Leishmann’s stain (Sigma-Aldrich). A minimum of 25 metaphase spreads were analyzed using the Metafer4 Complete Metafer System (MetaSystems, Altlussheim, Germany) with a Carl Zeiss AxioImager Z2 microscope (Carl Zeiss, Inc., Jena, Germany) and Ikaros karyotyping platform software (MetaSystems).

### Quantitative real-time PCR for *hTERT*

eSCs (P3; *n* = 6) and endometrial adenocarcinoma-derived Ishikawa cells (gifted from Cancer Center Karolinska University Hospital, Solna, Sweden) were lysed and total RNA extracted using the *Quick*-RNA Microprep Kit (Zymo Research, CA, USA) according to the manufacturer’s instructions. RNA was reconstituted in RNase free water, and concentrations were established using the Qubit RNA HS Assay Kit (Thermo Fisher Scientific). cDNA was produced using the SuperScript™ Vilo™ cDNA Synthesis kit (Thermo Fisher Scientific). Quantitative PCR (qPCR) was performed with the *hTERT* primer pair, FW: 5′GCCGTACATGCGACAGTTC3′ REV: 5′TCATTCAGGGAGGAGCTCTG3′ using Power SYBR® Green PCR Master Mix (Thermo Fisher Scientific) on a StepOnePlus™ Real-Time PCR System (Thermo Fisher Scientific) as per the manufacturer’s instructions. Ribosomal protein L13a RPL13A was used as a housekeeping gene FW: 5′CCTGGAGGAGAAGAGGAAAGAGA3′ REV: 5′TTGAGGACCTCTGTGTATTTGTCAA3′ [31].

### Quantitative Telomeric Repeat Amplification Protocol (qTRAP)

eSC telomerase activity was evaluated at P4–5 using qTRAP as previously described [12]. eSCs (0.5 E+06; *n* = 6) were washed in PBS, centrifuged at 500×*g* for 5 min, and resuspended in 100 μl Chemicon™ TRAPeze™ 1X CHAPS Lysis Buffer (EMD Millipore, Burlington, USA). Cells were incubated on wet ice for 30 min to ensure complete lysis. Lysates were centrifuged at 18,000×*g* for 20 min at 4 °C and the supernatant collected and snap frozen. A mastermix containing 5 μl (equivalent of 10,000 cells) of cell lysate, 100 ng of TS primer (5′AATCCGTCGAGCAGAGTT3′), 50 ng of ACX primer (5′GCGCGG[CTTACC]_3_CTAACC-3′), 12.5 μl of Fast SYBR™ Green Master Mix, and PCR grade water was made, with a reaction volume of 20 μl. qTRAP was performed using a StepOnePlus™ Real-Time PCR System, with reaction conditions of 25 °C for 25 min, 95 °C for 10 min, followed by 40 cycles of 95 °C for 15 s and 60 °C for 1 min and 30 s. A standard curve was run using the Ishikawa cell line (0–10,000 cells/reaction). Heat inactivated controls (85 °C, 30 min) acted as negative controls. Telomerase activity in cell lines or samples was calculated based on the threshold cycle. Data are expressed as relative telomerase expression (RTA) when compared to the Ishikawa cell control.

### Soft-agar tumorigenicity assay

eSCs (P5; *n* = 5) were checked for neoplastic transformation capacity (anchorage independent growth) using the CytoSelect™ 96-Well Cell Transformation Assay (Cell Biolabs, San Diego, USA) as per the manufacturer’s instructions. Ishikawa cells were expanded under the same conditions and treated as a positive control for anchorage independent growth. Briefly, in a 96-well plate, a 0.6% (w/v) base agar layer was added to the wells and allowed to solidify at 4 °C for 30 min. The second layer of 0.4% (w/v) agar containing eSCs or Ishikawa cells (1, 5, and 10 E+03 cells/ml) was added and incubated at 4 °C for 15 min. One hundred microliters of complete culture media was added to all wells, and the plate was incubated for 10 days at 37 °C in a humidified incubator at 5% CO_2_. To terminate the study and visualize the colonies, 50 μl of the provided agar solubilizing solution was added to each well and the plate was incubated at 37 °C for 60 min. The gels were aspirated up and down using a pipette to ensure solubilization, and 25 μl of the provided lysis buffer was added. Following 15 min incubation at room temperature, 10 μl from each well was transferred to a 96-well black microplate (Sigma-Aldrich) for relative fluorescence unit (RFU) at 485/520 nm using a FLUOstar® Omega plate reader (BMG Labtech, Germany). Cultures were assessed at time 0 (immediately after agar solidification, to determine RFU for each seeding cell number) and at day 10.

### Endometrial stromal cell responsiveness to inflammatory cytokines

eSCs (P4; *n* = 6) were exposed to 10 ng/ml human recombinant tumor necrosis factor alpha (TNFα; PeproTech Nordic, Stockholm, Sweden) and 100 IU/ml interferon gamma (IFNγ; Sigma-Aldrich) for 3 and 7 days in complete media, as previously described [32]. Complete media alone served as a control. Media was changed at day 4 for the day 7 stimulations to ensure equal conditioning of the media in downstream assays. Conditioned media was collected, centrifuged at 500×*g* for 5 min to remove cellular debris, snap frozen, and stored at − 80 °C prior to usage. The cells were detached using 0.05% (v/v) Trypsin-EDTA and counted for normalization of data.

#### Flow cytometry

eSCs were stained for cell surface markers CD120a, CD120b, CD119, HLA I, and HLA II for 15 min at room temperature in the dark (see Table 1 for antibodies). Stained cells were washed with PBS and resuspended in PBS/0.1% (w/v) BSA for analysis. LIVE/DEAD™ Fixable Aqua Dead Cell Stain Kit was used to assess viability. Twenty thousand gated (viable cells) events were recorded using a CytoFlexS and analyzed using FlowJo software.

#### Conditioned medium analyses

Enzyme-linked immunosorbent assay (ELISA): Interleukin (IL)-6 was measured within the cell culture supernatant using the Human IL-6 DuoSet ELISA (R&D Systems, Abingdon, UK) according to the manufacturer’s instructions. Prostaglandin E2 (PGE2) was assessed using the Prostaglandin E_2_ Parameter Assay Kit (R&D Systems) as per the manufacturer’s instructions.

Indoleamine 2,3-dioxygenase (IDO) activity was determined by measurement of L-kynurenine concentration, a terminal breakdown product of tryptophan, within the eSC-conditioned media [33, 34]. Briefly, 75 μl of 30% (v/v) trichloro-acetic acid (Sigma-Aldrich) was added to 150 μl of conditioned culture media and centrifuged at 8000×*g* for 5 min. The sample was combined with an equal volume of Ehrlich’s reagent (2% [w/v] p-dimethylbenzaldehyde in acetic acid), and the absorbance was measured at 490/492 nm using a Varioskan™ Flash Multimode Plate Reader (Thermo Fisher Scientific). Data were normalized to cell number.

### Co-culture of peripheral blood mononuclear cells and endometrial stromal cells

Peripheral blood mononuclear cells (PBMCs; *n* = 2 donors) were isolated from buffy coats as previously described [32]. Briefly, the blood was diluted with PBS and layered over Lymphoprep™ (Fresenius Kabi Norge, Oslo, Norway) and centrifuged at 500×*g* for 30 min. The PBMC fraction was removed, washed, and resuspended in RPMI (Thermo Fisher Scientific), 10% (v/v) human AB serum, 100 IU/ml penicillin, and 0.1 mg/ml streptomycin. PBMCs were labeled with the CellTrace™ CFSE Cell Proliferation Kit (Thermo Fisher Scientific) at a working concentration of 0.25 μM. PBMCs were activated with anti-CD2, anti-CD3, and anti-CD28 activation beads at a 1:2 bead to cell ratio (Miltenyi Biotec). Unloaded beads served as an unstimulated control. eSCs (*n* = 6; P3–4; 1E+05) were cultured with 1E+06 PBMCs, either in direct contact or separated by 0.4 μm polyethylene terephthalate transwells (BD Biosciences, CA, USA), for 5 days at 37 °C/ 5% CO_2_. PBMCs were stained at the end of the culture period for cell surface markers CD3, CD4, CD25, CD27, and CD45RA (see Table 1 for antibodies) for 15 min at room temperature. Stained cells were washed with PBS and resuspended in PBS/0.1% (w/v) BSA for analysis. LIVE/DEAD™ Fixable Aqua Dead Cell Stain Kit was used to assess viability. Fifty thousand viable, gated events were recorded using a CytoFlexS flow cytometer and analyzed using FlowJo.

### Statistical analysis

Comparisons in the groups were analyzed using one-way ANOVA with paired two-tailed *t* test. Mann Whitney *U* test or Wilcoxon test was used where data was non-parametric. Normality was determined by the Shapiro-Wilk test. Equal variance was determined by Bartlett’s Test and *F* Test when assuming Gaussian distribution. Statistical significance was assumed at *p* < 0.05 (Prism 8.0; GraphPad, San Diego, CA, http://www.graphpad.com).

## Results

### eSCs can be reliably isolated and expanded from healthy women

**Fig. 1:**
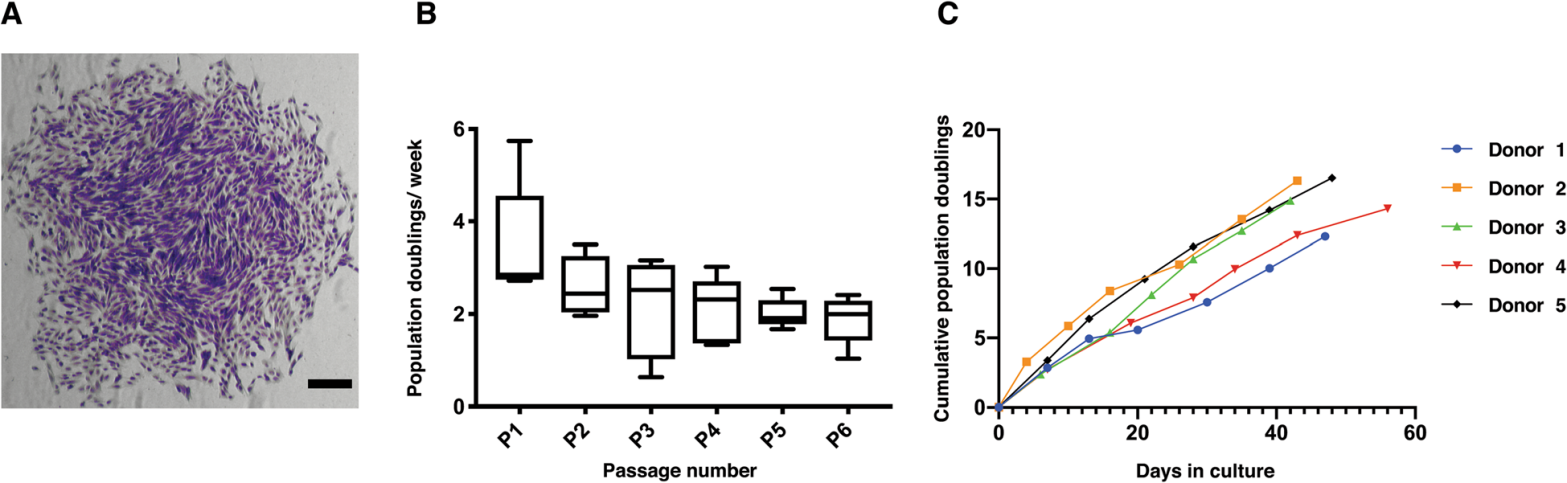
Endometrial stromal cells (eSCs) can be reproducibly isolated and expanded in vitro*.* **a** Example of an eSC colony at passage (P)1, stained with crystal violet, scale bar = 100 μm. **b** Mean population doubling rate per week for eSCs at P1–P6. Box and whisker plots indicate minimum to maximum range (*n* = 5 donors). **c** Cumulative population doublings for 6 consistent passages (*n* = 5 donors)

eSCs were isolated from 6 healthy, proven-fertile donors (age range 24–32), during the proliferative phase of the menstrual cycle (CD 7–9), and compared with regard to their ability to form colonies (Fig. 1a and Table 2). At P1, the average PD rate/week of the eSCs was 3.49, with a standard deviation (SD) of 1.28 (Fig. 1b). Cell yield at P1 and P2 was calculated as fold change compared to the original seeding density. At these passages, the approximate fold increase was 7 (7.42 fold at P1 and 7.1 fold at P2). This did not alter significantly between P1 and P6 (Fig. 1b). Cumulative growth kinetics supported this finding, demonstrating that all donors could be expanded for a minimum of 6 passages without entering replicative senescence (defined as 3 consecutive passages with < 0.5 PD/week; Fig. 1c).

**Table 2:** Endometrial stromal cell donor information

Donor	Cycle day	Age	% CFU-F at P1	Yield at P1 (fold change)	Yield at P2 (fold change)
1	7	29	15%	5.5	6
2	9	31	9%	7.2	4.3
3	8	31	19%	9.7	5.9
4	9	24	19%	5.2	8.1
5	7	31	17%	10.3	8
6	9	32	24%	6.6	10.3

Endometrial stromal cell (eSC) donor information including age, menstrual cycle day of cell isolation, colony forming unit-fibroblast (CFU-F), and fold change in yield of eSCs at passage (P) 1 and 2

### eSCs exhibit an MSC cell surface expression profile and show multi-lineage differentiation potential

**Fig. 2:**
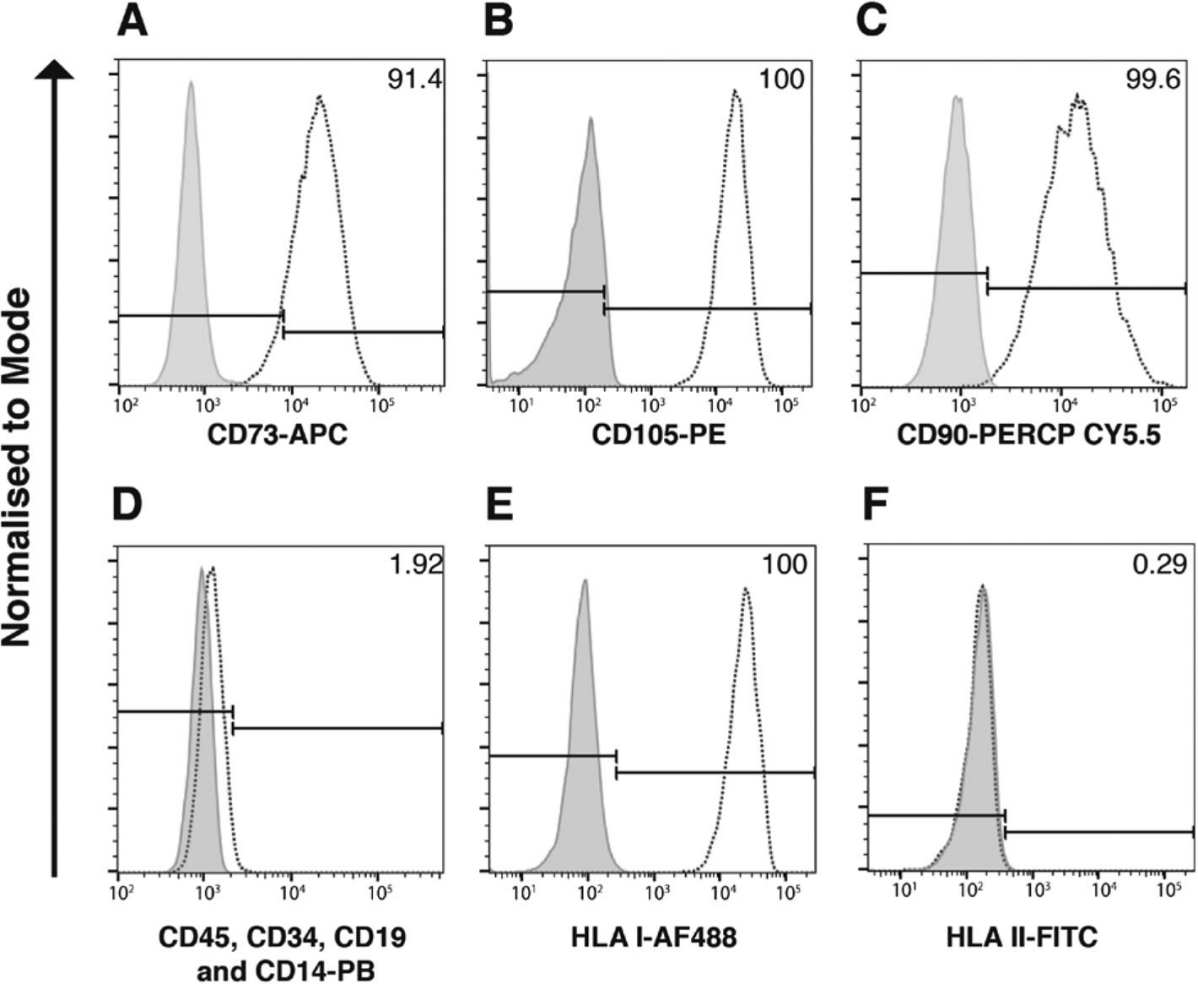
Endometrial stromal cells (eSCs) express typical mesenchymal stromal cell (MSC) markers. Representational histograms (colored white) of eSC cell surface expression of **a** CD73—allophycocyanin (APC); **b** CD105—phycoerythrin (PE); **c** CD90—peridinin chlorophyll protein (PerCP) -Cy5.5; **d** CD45, CD34, CD19, and CD14—Pacific Blue (PB); **e** human leukocyte antigen (HLA) I—Alexa Fluor (AF) 488; and **f** HLA II—fluorescein isothiocyanate (FITC; *n* = 6) with % of positive cells for each antigen. Gray histograms indicate respective immunoglobulin controls

Culture expanded eSCs were phenotypically characterized for their expression of cell surface markers CD90, CD73, CD105, CD45, CD34, CD14, CD19, HLA I, and HLA II, as per the ISCT’s minimal criteria for MSC [28]. eSCs from all donors were positive for CD90, CD73, CD105, and HLA I (> 95%; Fig. 2a–c and e) and were negative for CD45, CD34, CD19, CD14, and HLA II (> 2%; Fig. 2d, f). All donors were confirmed as possessing the ability to differentiate down both osteoblastic and adipocyte lineages upon stimulation, as we have previously demonstrated [35]. *Alizarin* Red staining confirmed the presence of calcium deposits associated with osteogenic differentiation, and adipocyte differentiation was evidenced by the detection of lipid droplets stained red with Oil Red O. No spontaneous differentiation was seen within the control wells (Additional file 1: Figure S1).

### eSCs retain chromosomal stability and demonstrate low tumorigenic potential upon in vitro expansion

**Fig. 3:**
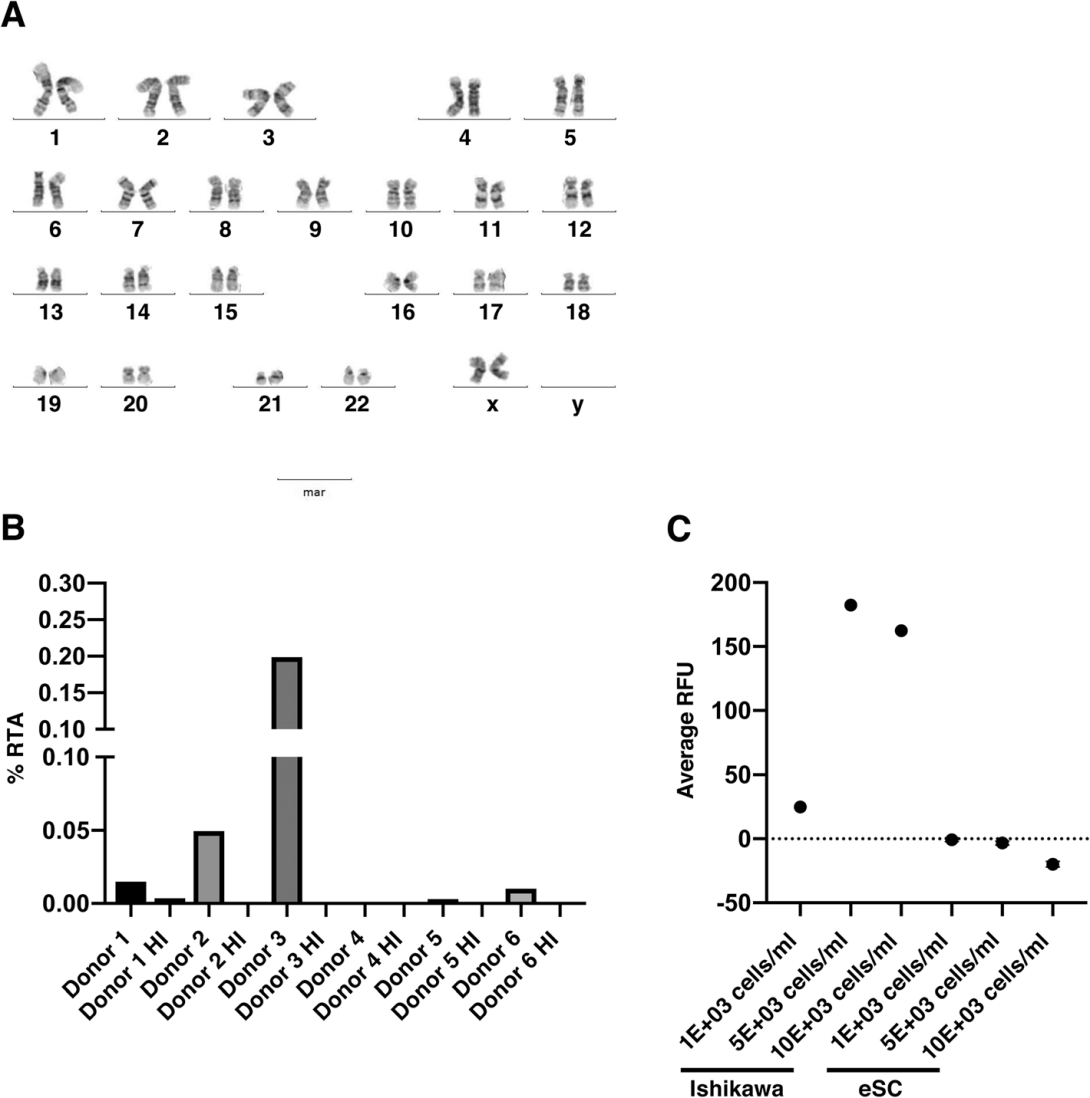
Endometrial stromal cells (eSCs) are genetically stable and exhibit low tumorigenic potential. **a** Representative normal karyotype (46,XX) of eSCs at passage (P)3 (*n* = 4). **b** Bar graph showing percentage relative telomerase activity (RTA), as assessed by quantitative telomeric repeat amplification protocol (qTRAP), in eSCs from each individual donor compared to the Ishikawa cancer cell line (*n* = 6). Heat inactivation (HI) was used as a negative control. **c** Bar graph of average relative fluorescence units (RFU) quantifying cell number as measurement of eSC and Ishikawa cell anchorage independent growth in a semi-solid agar. eSC data are expressed as mean RFU ± standard deviation of the mean (*n* = 5)

eSCs at P3, the point of expansion where a therapeutic dosage for localized therapy could theoretically be generated, was karyotyped to determine chromosomal stability. All karyotypes were categorized as normal (46,XX) with no aneuploidy, deletion/additions, or translocations (Fig. 3a). eSCs demonstrated extremely low levels of endogenous telomerase activity, with a mean RTA of 0.036% compared to Ishikawa cells. Heat inactivated controls confirmed the specificity of the reaction (Fig. 3b). These data were supported by detection of *hTERT* mRNA only in the Ishikawa positive control, and not in the eSCs (data not shown).

The soft agar tumorigenicity assay was utilized to investigate the potential anchorage-independent tumor forming capacity of eSCs. Tumor formation was observed within the Ishikawa positive control cultures, with the mean RFU increasing after 10 days at all 3 initial seeding densities (1000 cells/ ml = 24.92; 5000 cells/ ml = 182.4 and 10,000 cells/ml = 162.5; Fig. 3c). In contrast, the RFU of the eSCs did not increase relative to the original seeding density (1000 cells/ ml = − 0.73; 5000 cells/ ml = − 3.32 and 10,000 cells/ ml = − 19.81; Fig. 3c).

### eSCs constitutively express receptors for pro-inflammatory cytokines and are responsive to anti-inflammatory licensing

**Fig. 4:**
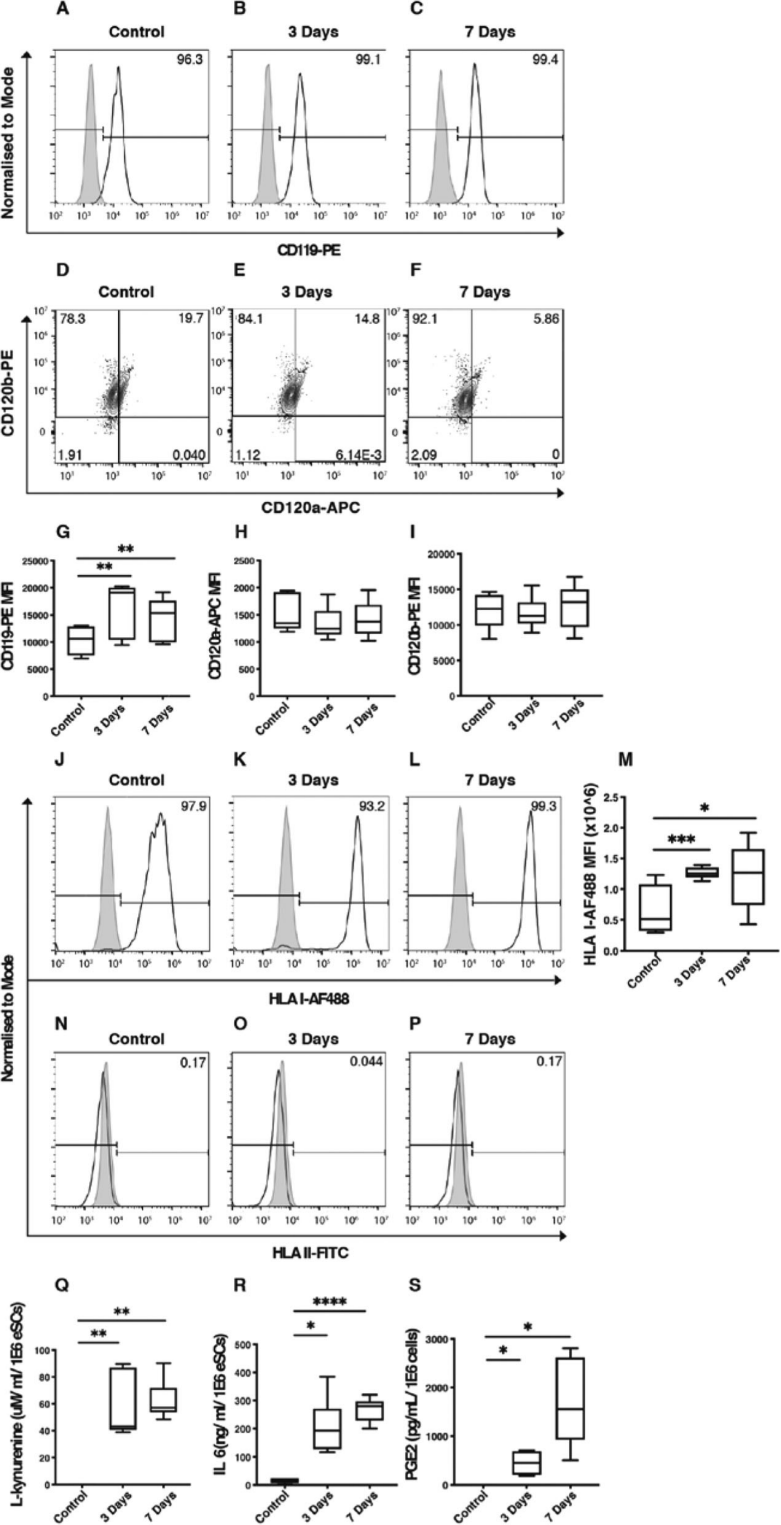
Endometrial stromal cells license to an anti-inflammatory phenotype without upregulation of human leukocyte antigen II. Representative histograms (colored white) of eSC cell surface expression of CD119 at **a** baseline, **b** 3 days, and **c** 7 days of interferon (IFN)γ and tumor necrosis factor (TNF)α exposure. Gray histograms indicate respective immunoglobulin controls. Representative scatterplots of eSC cell surface expression of CD120a and CD120b at **d** baseline, **e** 3 days, and **f** 7 days of IFNγ and TNFα exposure. Box and whisker plots showing the minimum and maximum distribution of the median fluorescence intensity (MFI) of **g** CD119—phycoerythrin (PE; *n* = 5), **h** CD120a—allophycocyanin (APC; *n* = 5), and **i** CD120b-PE (*n* = 5). Representative histograms (colored white) of eSC cell surface expression of human leukocyte antigen (HLA) I at **j** baseline, **k** 3 days, and **l** 7 days of IFNγ and TNFα exposure. Gray histograms indicate respective immunoglobulin controls. **m** Box and whisker plots showing the minimum and maximum distribution of the MFI of HLA I—Alexa Fluor (AF) 488 (*n* = 5). Representative histograms (colored white) of eSC cell surface expression of HLA II at **n** baseline, **o** 3 days, and **p** 7 days of IFNγ and TNFα exposure (*n* = 5). Gray histograms indicate respective immunoglobulin controls. Box and whisker plots showing the minimum and maximum distribution of baseline and post-licensing concentrations of **q** L-kynurenine (as a marker of indoleamine-2,3 dioxygenase activity), **r** interleukin (IL)-6, and **s** prostaglandin (PG) E2 in conditioned cell culture media (*n* = 6). *P < 0.05; **P < 0.01; ***P < 0.001; ****P < 0.0001

Expression of IFNγ receptor (R) I (CD119) and TNFRs I (CD120a) and II (CD120b) were evaluated on the surface of eSCs. eSCs demonstrated constitutive expression of CD119 and CD120b on the cell surface, with a subpopulation of CD120b+ eSCs also expressing CD120a (mean 35.25% ± SD 18.27%; Fig. 4a–f). CD119 expression increased significantly following stimulation at day 3 and day 7 compared to unstimulated controls (Fig. 4g; day 3 *p* = 0.0086; day 7 *p* = 0.0085). No change in expression level in response to prolonged inflammation was seen in CD120a and CD120b as assessed by median fluorescence intensity (MFI; Fig. 4h, i). Cell surface levels of the constitutively expressed marker HLA I were significantly increased following stimulation at day 3 and day 7 compared to unstimulated controls (Fig. 4m; day 3 *p* = 0.0004; day 7 *p* = 0.0241). eSCs expressed no HLA class II on their cell surface, and this was maintained following stimulation for both 3 and 7 days (Fig. 4n–p).

IDO production was switched on with L-kynurenine metabolites evident within the conditioned media only after 3 and 7 days of stimulation (Fig. 4q; *p* = 0.0022). No further increase in IDO activity was detected with prolonged (7 day) stimulation compared to 3 days (Fig. 4q). IL-6 was constitutively secreted by the eSCs, with stimulation significantly increasing levels detected at days 3 and 7 compared to controls (Fig. 4r; day 3 *p* = 0.0108; day 7 *p* < 0.0001). PGE2 was undetectable in control samples, but switched on following 3 and 7 days of stimulation (Fig. 4s; day 3 *p* = 0.0286; day 7 *p* = 0.0159).

### eSCs suppress the proliferation of CD4+ T cells and modulate their differentiation

**Fig. 5:**
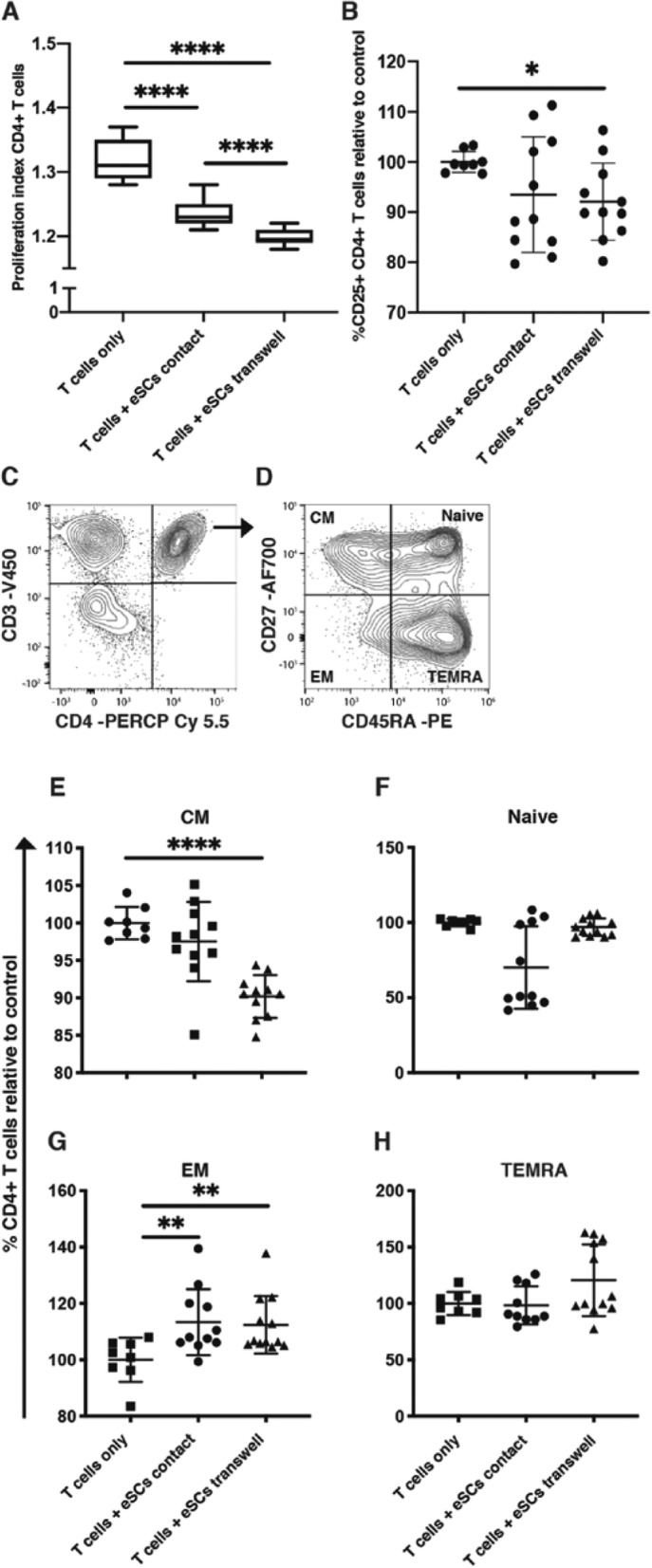
Endometrial stromal cells (eSCs) modulate the proliferation, activation, and differentiation status of CD4+ T cells. **a** eSCs suppressed the proliferation of CD4+ T cells in direct contact and transwell culture systems. Box and whisker plots showing the minimum and maximum distribution of the proliferation index of CD4+ T cells. **b** Scatter plot showing suppression of activation (CD25+) in CD4+ T cells by eSCs in transwell cultures (mean ± standard deviation of the mean [SD]). **c**, **d** Representative gating strategy to determine CD3+CD4+ T cells and their differentiation status based on expression of CD27 and CD45RA. **e** Scatter plot showing the significant drop in CD27+CD45RA- central memory (CM) CD4+ T cells with eSC exposure in transwell culture (mean ± SD). **f** Scatter plot showing no effect on the relative percentage of CD27+CD45RA+ naive CD4+ T cells with eSC exposure (mean ± SD). **g** Scatter plot demonstrating the significant increase in relative percentages of CD27-CD45RA- effector memory (EM) CD4+ T cell with eSC exposure in both direct contact and transwell cultures (mean ± SD). **h** Scatter plot showing no effect on the relative percentage of CD27-CD45RA+ effector memory T cells re-expressing CD45RA (TEMRA) CD4+ T cells with eSC exposure (mean ± SD). (*n* = 12) *P < 0.05; **P < 0.01; ****P < 0.0001

eSCs significantly suppressed the proliferation of CD4+ T cells, as assessed by proliferation index, both in direct contact and under transwell culture conditions (Fig. 5a; *p* < 0.0001). This suppression was significantly more pronounced in transwell compared to direct cell to cell contact (Fig. 5a; *p* < 0.0001). T cell activation, as assessed by the expression of CD25, was significantly suppressed only within transwell cultures compared to PBMC only controls (Fig. 5b; *p* = 0.0121). Further analysis on the regulatory T cell fraction (CD4+CD25+CD127-) suggested no effect by eSCs under either co-culture condition (data not shown).

Exposure of PBMCs to eSCs differentially modulated the status of CD4+ T cells under transwell and direct co-culture conditions. The number of CD4+CD27+CD45RA- central memory (CM) T cells was significantly reduced only in transwell eSC cultures compared to PBMC only controls (Fig. 5e; *p* < 0.0001), while the number of CD4+CD27-CD45RA- effector memory (EM) T cells was increased in both direct contact (Fig. 5g; *p* = 0.0053) and transwell (Fig. 5g; *p* = 0.0058) eSC co-cultures compared to PBMC only controls. No changes were observed in the CD4+CD27+CD45RA+ naïve and CD4+CD27-CD45RA+ effector memory T cells re-expressing CD45RA (TEMRA) fractions with eSC exposure (Fig. 5f, h).

## Discussion

The potential for culture-expanded MSC populations, especially bone marrow-derived MSCs, to be utilized as an advanced therapeutic medicinal product in the treatment of numerous disorders has been extensively reported [36]. The breadth of MSC therapy has evolved over the last decade, with a growing interest in the development of cell therapy for fibrotic diseases, such as AS. With new knowledge there has been an overhaul of regulatory requirements for cell therapy, and a growing appreciation that MSCs from different tissue sources possess unique properties as a result of their role in the in vivo niche. The aim of this study was therefore to establish protocols for the reliable isolation and expansion of eSCs, and establish their stability and immunomodulatory profiles.

Here we report that isolation and expansion of eSCs from healthy donors are reproducible, generating a stromal population that maintains genetic stability over multiple passages (sufficient to generate a clinical cell dose for local administration to the endometrium), and in keeping with the minimum phenotypic characterization outlined by the ISCT [28]. However, investigation into the responsiveness of these cells to inflammatory stimuli, and their ability to modulate adaptive immune cell subsets, demonstrated distinguishing features, separating eSCs from other adult MSC sources.

Our isolation protocol is a streamlined version of a previous endometrial isolation protocol [37], retaining a heterogeneous stromal compartment, as characterized in recent single-cell sequencing findings [25], which ensures high cell viability with maximum cell retention from pipelle biopsies. Using the ISCT MSC guidelines [28] as a reference to characterize our cell product, we found ex vivo expanded eSCs adhered to plastic; had positive (> 95%) cell surface marker expression for CD105, CD73, CD90, and HLA I; and lacked expression of hematopoietic surface antigens CD45, CD34, CD14, CD19, and HLA II (< 2%). As previously reported, eSCs showed multipotent differentiation potential; however, small variations/ predispositions in the degree of differentiation were observed between donors. In line with existing knowledge of the stroma being composed of heterogeneous subpopulations, different proliferation rates, cell morphologies, immunophenotype, and multi-differentiation potential can all be expected [38–41]. This is an important consideration in developing cell therapy products and should be taken into consideration with regard to the expected therapeutic mode of action. Knowledge regarding the potential mode of action of stromal cells in the treatment of endometrial disorders, such as AS, remains scarce, and therefore, we made the informed choice to not narrow down the cell product to a specific subpopulation, e.g., the endometrial pericyte: platelet-derived growth factor receptor beta (PDGF-Rβ)+/CD146+ or Sushi Domain Containing 2 (SUSD2)+, as we cautiously cannot exclude that the heterogeneous composition would be part of the therapeutic effect [3, 22, 42, 43].

Isolated eSCs were expanded in xeno-free culture media, with no significant changes in growth rate between P1 and P4; the hypothesized passage ranges for cell therapy being P1–3. Some variability was seen between donors in cumulative PDs at later passages (P4–6), which may be indicative of donor to donor variability. As any product generated will require ex vivo manipulation, in the form of culture expansion, it was important to determine the genetic stability of the eSCs and evaluate the risk of tumorigenicity [44]. Karyotyping our eSCs at P3 showed no chromosomal abnormalities. Likewise, eSCs were unable to show anchorage independent growth in the soft agar colony formation assay and exhibited low telomerase enzyme activity, with no mRNA expression of *hTERT*, features indicative of tumorigenic cells. Similarly, in a previous study investigating telomerase in the proliferative phase endometrium, activity and gene expression were only localized in the glandular epithelium [45]. This falls in line with the majority of MSC studies that have seen no evidence of genomic instability at early passages [40, 46–48] or tumorigenic potential [48].

In recent years, it has been established that MSCs contribute to tissue regeneration predominantly through the actions of their secretome [18]. Recommendations from ISCT [29, 49] have stressed the importance of understanding how stromal cells may reduce inflammation and modulate the innate and adaptive immune systems. Thus, we have studied the responsiveness of the eSCs to pro-inflammatory cytokines to determine the homeostatic state of our cells, and how responsive eSCs are to a pro-inflammatory environment as might be seen in AS. eSCs constitutively expressed the predominant receptor for IFNγ (CD119) and TNFα receptor II (CD120b). CD119 expression was upregulated by the presence of pro-inflammatory cytokines in culture, while expression of CD120a and b remained unchanged.

As seen for other MSC sources, IFNγ and TNFα licensed the eSCs to an anti-inflammatory state. HLA class I on the surface of the cells was significantly increased, a phenomenon previously demonstrated for both adult and fetal MSCs [34, 50, 51]. In contrast to these reports, eSCs showed no expression of HLA II on the cell surface following 7 days of pro-inflammatory stimulation. These findings demonstrate that eSCs have tissue-specific properties, which have not been previously reported in other cell sources. Embryonic trophoblastic cells have shown a similar immunologic phenotype, and it could be postulated that this mechanism may contribute to fetal-maternal immune tolerance [52]. In the current wave of research into allogeneic induced pluripotent stem cell (iPSC) therapies with HLA I and II knockouts [53], as part of an effort to create a universal iPSC donor, it becomes clear that understanding the translational inactivation of HLA II seen in the endometrium and trophoblast cells could be valuable for many cell therapies, as well as, for transplant tolerance, therefore warranting further investigation at the (epi)genomic level.

Analysis of the eSC secretome demonstrated a switch to an immunomodulatory/suppressive profile, with the production and upregulation of IDO, IL-6, and PGE2 in eSC spent media. This change in secretory profile has been previously reported in other stromal cell populations and is known to mediate the suppression of T, NK, and dendritic cells [54–56]. Furthermore, the secretion of PGE2 by MSCs has been reported to promote the proliferation of epithelial cells, and therefore, their roles in immunomodulation and healing have been hypothetically linked [57]. Such properties and secretion of key biomolecules lend eSCs to the development of novel cellular therapeutics for endometrial disorders, with elements of both immune and tissue repair dysfunction.

Within the context of the endometrium, the phenotypic profile of the cells may be reflective of the menstrual cycle day at sample collection, suggesting there could be plasticity in the responsiveness of the stroma to differing levels of pro-inflammatory signals dependent on cycle stage. Here biopsies were taken during the proliferative phase, suggesting that significant differences could be seen between this cell source and menSCs. PGE2, for example, is known to be abundant in the endometrium and regulates vasodilation and edema formation at menstruation [27]. This further highlights the importance of considering CD when taking biopsies for stromal cell isolation.

We further investigated the effects of eSCs and their secretome on the profile of stimulated T cells. As previously reported for MSCs, our results suggest eSCs suppress activated CD4+ T cell proliferation via cell-to-cell contact and paracrine factors [58–60]. The overall inhibitory effect being more pronounced in the transwell condition, matching the effect seen by Di Nicola et al. [59]. It has been previously documented that the predominant suppressive effect by MSCs on T cell proliferation occurs through the paracrine effects of PGE2 on monocytes [61]. Furthermore, it has been reported that IL-6, IDO, and PGE2 secreted by MSCs can skew both peripheral monocytes and tissue-resident macrophages to an M2, anti-inflammatory phenotype with elevated secretion of IL-10 [56, 62, 63]. Monocytes constitute around 10% of the PBMC fraction, and therefore, the induction of suppression by the eSCs may be an indirect effect through the monocyte population. In contrast, a suppression in the activation state of CD4+ T cells was only seen by the eSC secretome. This may be indicative of additional immunomodulatory pathways, including that of programmed cell death protein 1 (PD-1):PD-ligand(L)1/PD-L2 axis, demonstrated to directly regulate T cell CD25 expression by the bone marrow MSC secretome [32].

eSCs primarily elicited a decrease in CM T cells under eSC transwell co-culturing conditions and an increase in the relative expression of EM T cells in transwell and cell-to-cell contact co-culture conditions. MSCs derived from similar fetal/ maternal tissue, i.e., placenta and amniotic fluid, also significantly decrease memory T cell subsets, yet also induce an increase in naïve T cells, an effect we did not see in our co-culture models [64]. MSCs derived from other origins have been shown to suppress CM and EM CD4+ T cell differentiation and cytokine production, inducing a more immune tolerant profile [65–67]. Interestingly, it has been reported that IL-10 decreases both CM and EM development specifically within the CD4+ T cell compartment in response to viral infection [68]. The notion that we see an increase in EM T cells may be a link to their in vivo role. EM T cells are characterized by their lack of C-C chemokine receptor (CCR)7 expression, meaning that they are primed to leave the circulation and enter inflamed tissue to participate in reparative responses [69]. As EM T cells are the first line of defense against reinfection [70], with privileged access to tissue sites vulnerable to pathogens, such as the endometrium, it is not surprising that eSCs possess a directed response to this immune subset on seeing inflammation.

## Conclusions

In conclusion, we demonstrate that eSCs can be reproducibly isolated and expanded in vitro, with maintenance of genetic stability. eSCs, despite initial similarities to MSCs as defined by the ISCT, exhibit distinct differences in phenotype and function, in particular their mode of immunomodulatory action and HLA II expression, which are most likely adapted to suit the microenvironment of the endometrium. These findings warrant further investigation to establish their mode of immunomodulatory action and potential for therapeutic development in the treatment of endometrial disorders, such as AS.

## Supplementary information

**Additional file 1: Figure S1.** Confirmation of endometrial stromal cell potential. eSCs were subjected to osteogenic or adipogenic differentiation by culturing with induction or control medium. Osteogenic differentiation was detected by staining mineralized matrix with *Alizarin* Red. Representative photomicrographs of A) control, scale bar = 200 μm and B) induced cultures, scale bar = 200 μm. Adipogenic differentiation was detected by Oil red O staining of lipid rich vacuoles. Representative photomicrographs of C) control, scale bar = 100 μm and D) induced cultures, scale bar = 100 μm (*n* = 6).

## Data Availability

All data generated or analyzed during this study are included in this published article.

## Abbreviations

AF: Alexa Fluor
APC: Allophycocyanin
AS: Asherman’s syndrome
BSA: Bovine serum albumin
CCR: C-C chemokine receptor
CD: Cycle day
CFU-F: Colony-forming unit-fibroblasts
CM: Central memory
ELISA: Enzyme-linked immunosorbent assay
EM: Effector memory
eSC: Endometrial stromal cell
FITC: Fluorescein isothiocyanate
HLA: Human leukocyte antigen
IDO: Indoleamine 2,3-dioxygenase
IFNγ (R): Interferon gamma (receptor)
IL: Interleukin
iPSC: Induced pluripotent stem cell
ISCT: International Society for Cellular Therapy
MEMα: Minimum Essential Medium Eagle – Alpha Modification
MenSCs: Menstrual blood-derived stromal cells
MFI: Median fluorescence intensity
MSC: Mesenchymal stromal cell
P: Passage
PB: Pacific blue
PBMC: Peripheral blood mononuclear cells
PBS: Phosphate buffered saline
PD: Population doubling
PD-1: Programmed cell death protein 1
PDGFR: Platelet-derived growth factor receptor
PD-L: Programmed cell death protein 1 ligand
PE: Phycoerythrin
PerCP Cy5.5: Peridinin chlorophyll protein
PGE2: Prostaglandin E2
qPCR: Quantitative real-time polymerase chain reaction
qTRAP: Quantitative Telomeric Repeat Amplification Protocol
RFU: Relative fluorescence unit
RPMI: Roswell Park Memorial Institute
RTA: Relative telomerase activity
SUSD2: Sushi Domain Containing 2
TEMRA: Effector memory T cells re-expressing CD45RA
TNFα: Tumor necrosis factor alpha
UC MSC: Umbilical cord-derived MSC
