# Supplementary figures and images for: Endometrial stromal cells exhibit a distinct phenotypic and immunomodulatory profile
Source: Stem Cell Res Ther. 2020 Jan 6;11:15. doi: 10.1186/s13287-019-1496-2 (PMC6945659; doi:10.1186/s13287-019-1496-2)

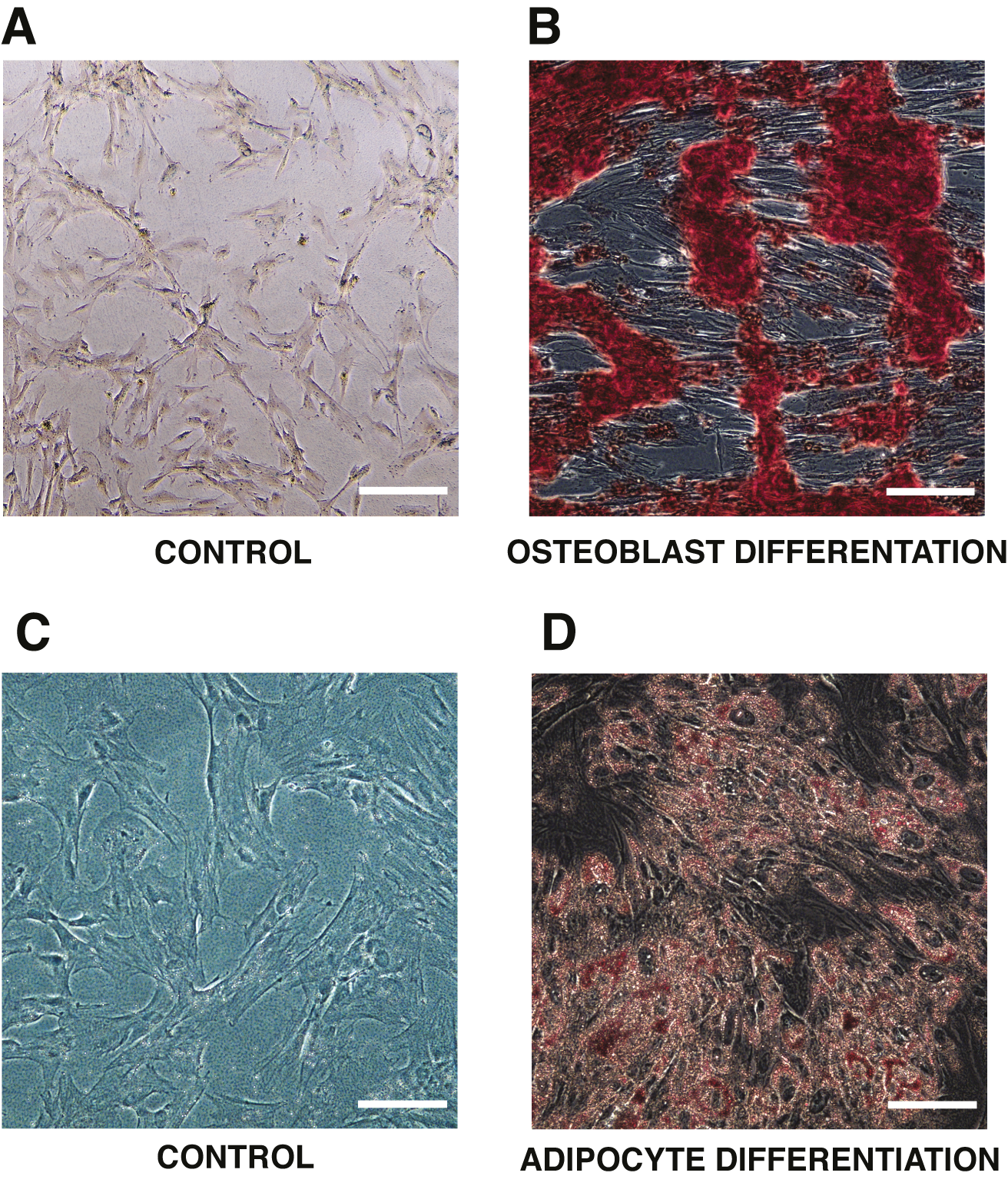

Supplement: Supplementary file 1 — Additional file 1: Figure S1. Confirmation of endometrial stromal cell potential. eSCs were subjected to osteogenic or adipogenic differentiation by culturing with induction or control medium. Osteogenic differentiation was detected by staining mineralized matrix with Alizarin Red. Representative photomicrographs of A) control, scale bar = 200 μm and B) induced cultures, scale bar = 200 μm. Adipogenic differentiation was detected by Oil red O staining of lipid rich vacuoles. Representative photomicrographs of C) control, scale bar = 100 μm and D) induced cultures, scale bar = 100 μm (n = 6). [file 13287_2019_1496_MOESM1_ESM.tif]
